# Supplementary material for: Characterization of the Nero Siciliano Pig Fecal Microbiota after a Liquid Whey-Supplemented Diet
Source: Animals (Basel). 2023 Feb 12;13(4):642. doi: 10.3390/ani13040642 (PMC9951753; doi:10.3390/ani13040642)
Supplement: Supplementary file 1 [file animals-13-00642-s001.zip › Table_S1_Environmental_parameters.pdf]

**Table S1.** Environmental parameters detected inside and outside the barn before (T0, May), and one (T1, June) and two (T2, July) months after the beginning of the study.

| Environmental parameters | Experimental period | | | | | |
|---|---|---|---|---|---|---|
| | T0 | | T1 | | T2 | |
| | Inside | Outside | Inside | Outside | Inside | Outside |
| Temperature (°C) | 21.5 | 22 | 24.5 | 25 | 26 | 26.5 |
| Relative humidity (%) | 61 | 62 | 61 | 62 | 53 | 55 |

The parameters reported are expressed as mean values
