# Supplementary figures and images for: Characterization of the Nero Siciliano Pig Fecal Microbiota after a Liquid Whey-Supplemented Diet
Source: Animals (Basel). 2023 Feb 12;13(4):642. doi: 10.3390/ani13040642 (PMC9951753; doi:10.3390/ani13040642)

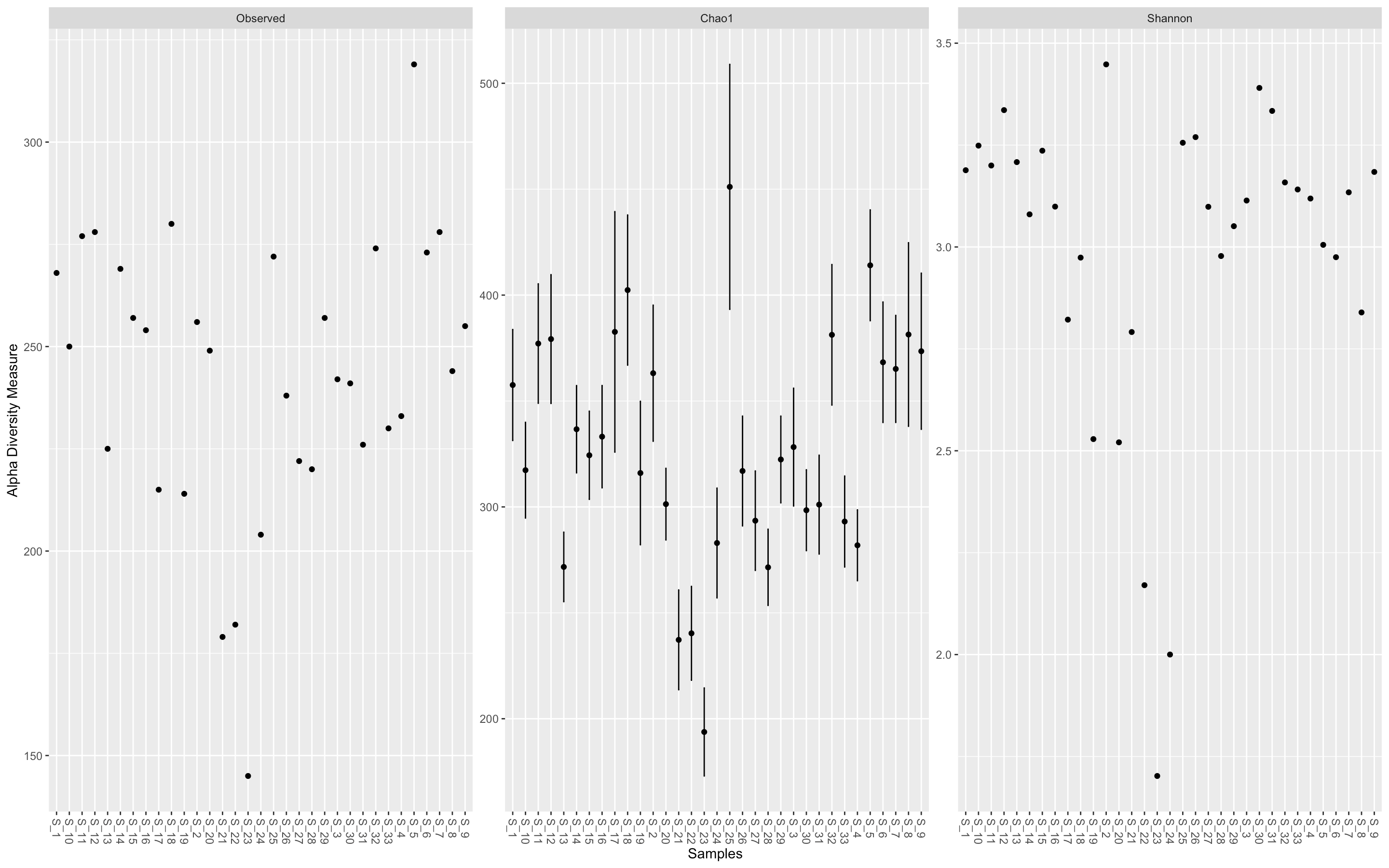

Supplement: Supplementary file 1 [file animals-13-00642-s001.zip › Figure_S1_alfa_diversity_plot.png]

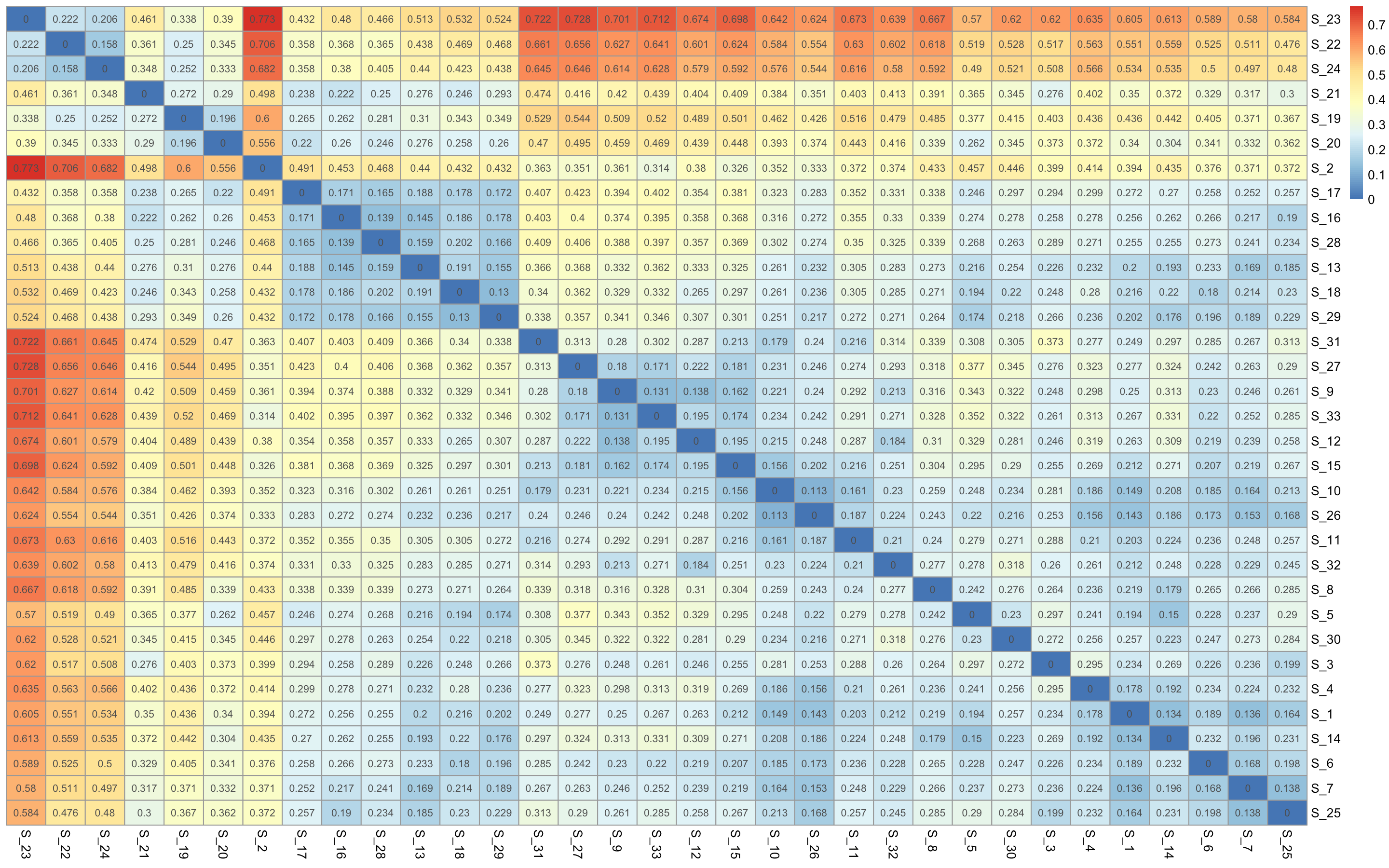

Supplement: Supplementary file 1 [file animals-13-00642-s001.zip › Figure_S2_beta_diversity_heatmap.png]

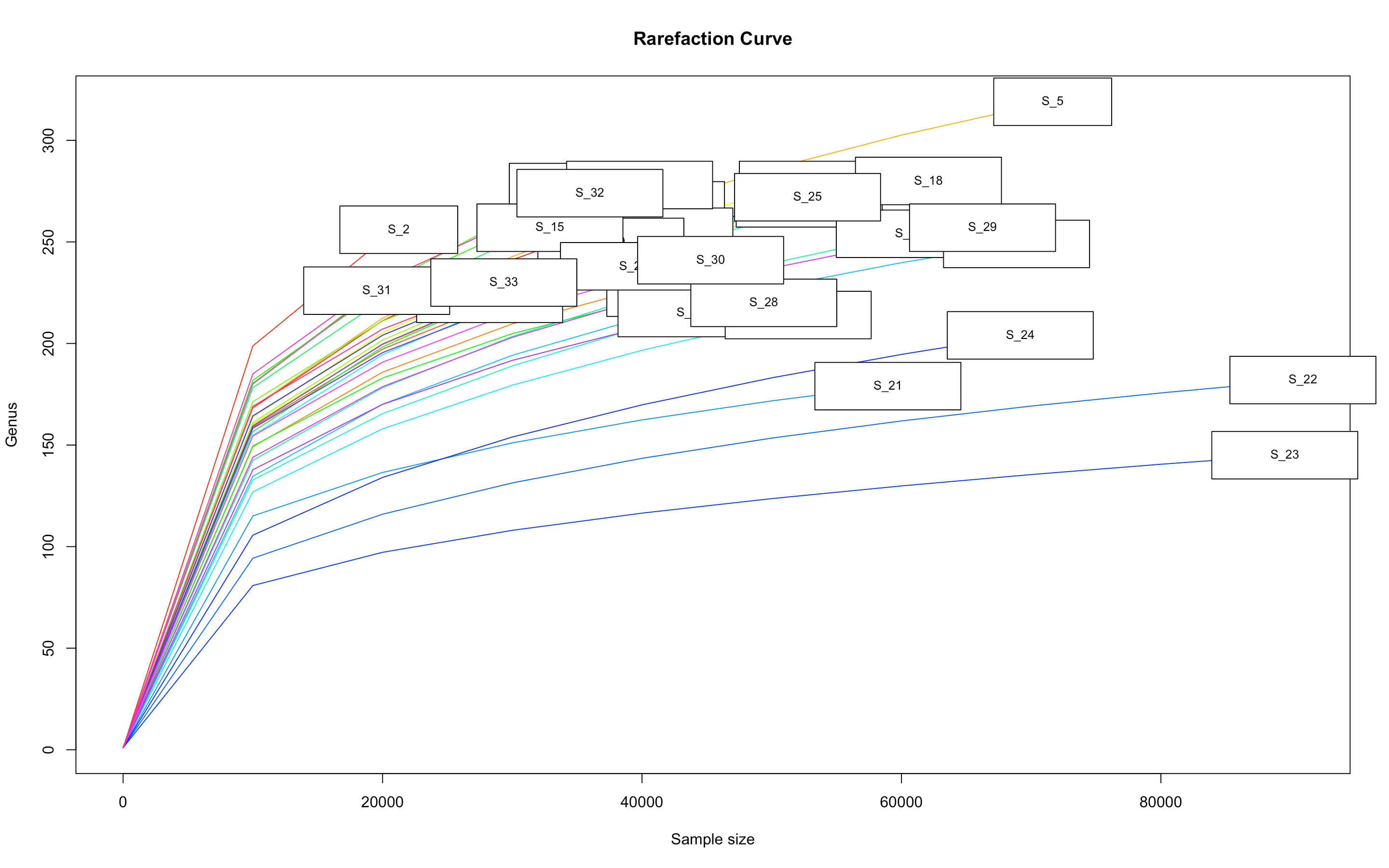

Supplement: Supplementary file 1 [file animals-13-00642-s001.zip › Figure_S3_Rarefaction_curve_graph.png]
